# Splicing Factors Have an Essential Role in Prostate Cancer Progression and Androgen Receptor Signaling

**DOI:** 10.3390/biom9040131

**Published:** 2019-04-01

**Authors:** Ken-ichi Takayama

**Affiliations:** 1 Department of Functional Biogerontology, Tokyo Metropolitan Institute of Gerontology, 35-2 Sakae-cho, Itabashi-ku, Tokyo 173-0015, Japan; ktakayama-tky@umin.ac.jp; Tel.: +81-3-3964-3241; 2 Department of Geriatric Medicine, Graduate School of Medicine, the University of Tokyo, 7-3-1 Hongo, Bunkyo-ku 113-0033, Japan

**Keywords:** prostate cancer, androgen receptor, splicing factor, PSF, NONO

## Abstract

Although inhibition of the androgen–androgen receptor (AR) axis effectively represses the growth of prostate cancer, most of all cases eventually become castration-resistant prostate cancers (CRPCs). Enhancement of the expression of AR and its variants along with the downstream signals is important for disease progression. AR-V7, a constitutive active form of AR, is generated as a result of RNA splicing. RNA splicing creates multiple transcript variants from one pre-messenger RNA (mRNA) by removing introns/exons to allow mRNA translation. The molecular mechanisms leading to marked increases of AR and generation of AR-V7 have been unclear. However, recent papers highlighted the roles of RNA splicing factors which promote AR expression and production of variants. Notably, a broad range of splicing components were aberrantly regulated in CRPC tissues. Interestingly, expression of various spliceosome genes is enhanced by RNA-binding protein splicing factor proline- and glutamine-rich (PSF/SFPQ), leading to changes in the expression of *AR* transcript variants. Moreover, inhibition of several splicing factors repressed tumor growth in vivo. Altered expression of splicing factors is correlated to biochemical recurrence in prostate cancer patients. Thus, these findings suggest that splicing factors would be a potential therapeutic target. This review focuses on the emerging roles of splicing factors in prostate cancer progression and AR signaling.

## 1. Introduction

Dysregulation of gene expression is a well-known feature of cancer cells [1]. Of note, messenger RNA (mRNA) splicing to remove introns from precursor messenger RNA (pre-mRNA) is a critical step in the post-transcriptional regulation of gene expression [2]. Most human genes generate multiple RNA transcripts through alternative splicing; this phenomenon enables cells to produce a variety of distinct proteins from a single gene [3]. An enzymatic complex in the nucleus, known as the spliceosome, is responsible for mRNA splicing. The spliceosome consists of five small nuclear ribonucleoproteins, called snRNPs (U1, U2, U4, U5, and U6) [4,5,6,7,8,9]. Each snRNP includes small nuclear RNA (snRNA) complexed to the related proteins, which are named splicing factors. Recent genomic sequencing studies identified that the mutation and aberrant expression of splicing factors are important mechanisms to generate the diversity of gene functions required for cancer progression. Therefore, I present a simplified summary of the spliceosome assembly pathway. In addition, several important reports have demonstrated that the androgen receptor (AR) splicing pathway is a key driver for prostate cancer progression [10,11,12,13]. Prostate cancer is the second leading cause of cancer deaths in men and the growth of prostate tumors is controlled by the androgen hormone [14,15]. In this review, I have presented an overview of the implication of the splicing machinery in the progression of prostate cancer.

## 2. The Role of the Androgen Receptor in Prostate Cancer Progression

**Figure 1:**
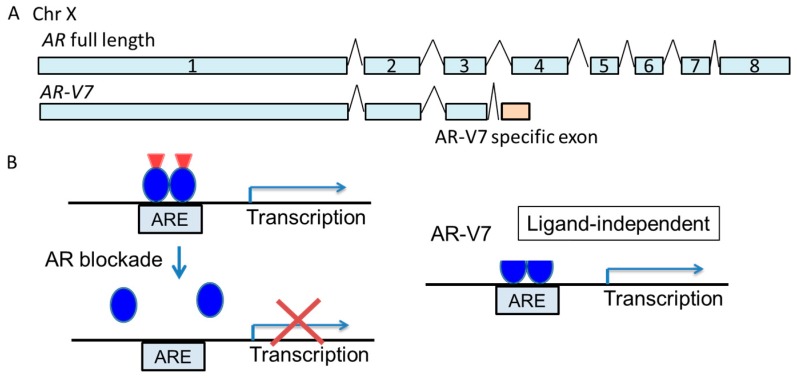
Genomic structure and transcriptional regulation by androgen receptor-variant 7 (AR-V7). A. A schematic of the genomic structure of the *androgen receptor (AR)* gene (exons 1–8) and the splicing variant, *AR-V7*. B. The AR-V7 protein lacks the ligand-binding domain of AR. Therefore, AR blockade by anti-AR drugs is not effective for tumors expressing AR-V7, because AR-V7 exerts ligand-independent transcriptional activity. ARE: androgen response element.

Because prostate cancer progression is dependent on androgen signaling, androgen depletion therapy (ADT) is the primary treatment for advanced prostate cancer [14,15]. Although ADT is initially effective for tumor regression, most cancers progress to lethal castration-resistant prostate cancer (CRPC), for which no curative therapy is available [15]. It is commonly proposed that activation of the AR signaling contributes to CRPC development through several mechanisms, such as AR overexpression, intra-tumoral androgen synthesis, induced expression of AR co-regulators, and alternative AR activation by cytokines and growth factors [16,17,18]. Interestingly, recent studies have shown that the AR is also expressed as C-terminal truncated variants, called AR-variants (AR-Vs), by an alternative RNA splicing process. AR-Vs lack the ligand-binding domain of full-length AR, constitutively activate AR-downstream transcription, and promote tumor growth, even under castrate conditions [10,11,12,13] (Figure 1). Although a number of AR-Vs have been identified in prostate cancer cell lines and xenografts, AR-V7 is the most commonly expressed AR-V in CRPC tissues [19]. *AR-V7* mRNA is spliced at the alternative 3’ splice site (3’SS) of a cryptic exon, exon 3B, rather than at the 3’SS of exon 4 [10]. However, it is still unclear how *AR-V7* mRNA is produced. It has been established that AR-V7 expression is well correlated with CRPC development and resistance to androgen deprivation therapy [19,20,21,22]. AR-V7 can be detected in circulating tumor cells from patients with CRPC. Patients with higher AR-V7 expression in circulating tumor cells had shorter prostate specific antigen (PSA) progression-free survival [13]. A recent meta-analysis of clinical studies reported that AR-V7 positivity was associated with higher PSA recurrence and poor prognosis in patients with CRPC treated with AR antagonists [20]. Although not every study has validated these relationships [23], these findings suggest that AR-V7 may serve as a prognostic and predictive biomarker for patients with metastatic prostate cancer.

Tumors that express canonical AR signaling are highly sensitive to ADT [24]. Patients with this phenotype of cancer have prolonged survival. Canonical AR signals drives the expression of genes associated with differentiation and luminal cell identity. In contrast, it has been shown that AR-Vs may bind to its unique targets, independently of full-length AR [25,26]. It has been demonstrated that the transient expression of exogenous AR-V7 preferentially modulated cell-cycle regulated genes to promote the mitotic phase and cause cell growth. Thus, those cell-division associated genes are unique AR-V targets that may reflect the proliferative events driven either by AR-Vs or androgen-stimulated AR. The cell-cycle regulator ubiquitin-conjugating enzyme E2 C (UBE2C), is a representative target of AR under androgen-depleted conditions [27]. The expression of cell-cycle regulatory gene UBE2C is specifically regulated by AR-V7 and not by AR-full length in both prostate cancer cells and tissues from patients with CRPC [28]. These effects contribute to cell proliferation, even if sustained by relatively low levels of AR-V7.

## 3. The Splicing Machinery for Regulating Gene Expression

Protein-coding exons are disrupted by non-coding introns [29]. Through the development of genome sequencing analysis, it has become clear that precursor messenger RNA (pre-mRNA) splicing can occur at a large scale and with biological complexity. Biochemical studies have demonstrated that the RNA cleavage and ligation reactions necessary for intron removal in protein-coding mRNAs occur in a large ribonucleoprotein (RNP) machine called the spliceosome [29]. Alternative splicing pathways result in the expansion of the human proteome and many physiological mechanisms [1,2,3]. One of the underlying rationales for the purification of discrete splicing complexes is to determine the structure of these defined intermediates along the splicing pathway. Because of the dynamic nature of the spliceosome, structural biologists have predominantly used electron microscopy to assess the structures of a variety of spliceosomal complexes, as well as isolated snRNPs [30,31]. 

**Figure 2:**
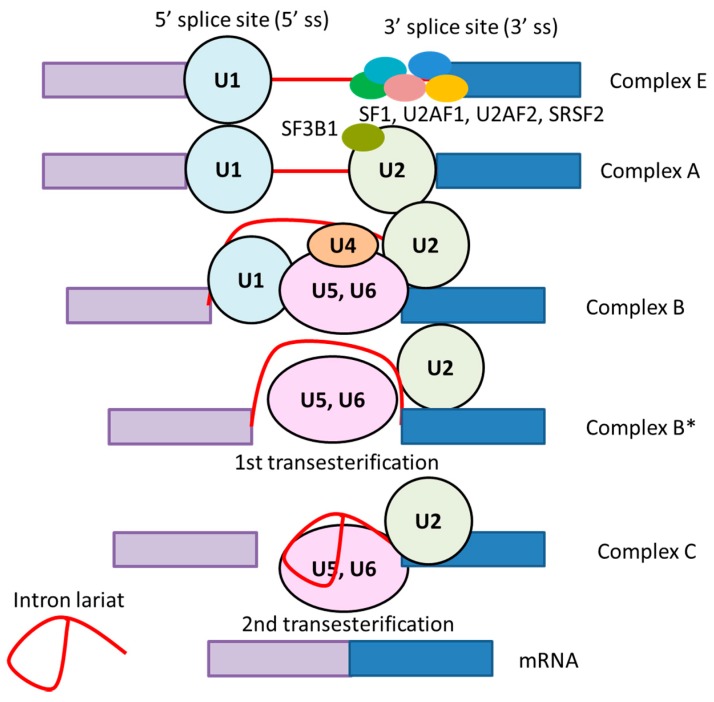
RNA splicing machinery that involves several distinct complexes. The first complex is formed by binding of U1snRNP and U2AF1/2 to the transcribed pre-mRNA. Establishment of complex E activates the recruitment of U2snRNP and forms complex A. The U4/U6.U5 tri-snRNP complex joins and leads to the formation of complex B. After release of U1/U4 snRNPs, complex B is catalytically activated (complex B*), followed by the next conformational change that results in the formation of complex C. Complex C subsequently catalyzes the second esterification reaction and excises the intron as intron lariat. snRNP: small nuclear ribonucleoprotein. U2AF: U2 small nuclear RNA auxiliary factor.

Mechanistically, both the regulatory RNA sequences and their correlated RNA splicing proteins are important for determining splicing site. Some regulatory sequences are called exonic splicing enhancers (ESEs) or intronic splicing enhancers (ISEs) [29]. Moreover, RNA splicing is closely associated with transcriptional process. Both transcription initiation and elongation rates affect splicing. The spliceosomal small nuclear ribonucleoproteins (snRNPs) (U1, U2, U4, U5, and U6) interact with the transcribed RNA stepwise (Figure 2) to effect the removal of an intron from a precursor messenger RNA (pre-mRNA) including exons [30]. Base pairing of snRNAs to conserved sequences on pre-mRNA, as well as interactions of numerous splicing accessory proteins and RNA–protein interactions, are critical in guiding the huge spliceosomal complex to the sites of pre-mRNA for splicing [29,30,31]. These sites can be either intronic or exonic and can be positive (splicing enhancers) or negative (splicing silencers). In addition to RNA-protein binding, RNA-RNA base pairing can define the splicing site [29]. Connective networks have been formed between chromatin modifications, RNA polymerase II speed, and alternative splicing patterns.

During transcription elongation, these protein complexes move along the gene. The RNA spliceosome complex screens the pre-mRNA to determine and excise the splice sites before the termination of transcription [32]. Thus, the expression of a specific splice variant is regulated by both gene transcription rate and the splicing factor binding to the pre-mRNA during the splicing process [2]. Recent progress in the field has demonstrated that the catalytic center of the spliceosome is also composed of RNA, so it has been confirmed that the spliceosome is a ribozyme, similar to the ribosome. In addition, investigations have isolated, purified, and characterized the protein composition and biochemical activities to determine the structures of several of these distinct forms of the spliceosome as they proceed along the reaction pathway [4,5,6,7,8,9].

Spliceosome assembly needs to occur repeatedly every time an intron is removed from a pre-mRNA in a eukaryotic nucleus [32]. An intron includes four consensus elements: (i) the 5’ splice site (5’ SS), which is located at the 5’end of the intron; (ii) the 3’ SS, which is located at the 3’ end of the intron; (iii) the branch point sequence (BPS), which is located upstream of the 3’ SS; and (iv) the polypyrimidine tract, which is located between the BPS and the 3’ SS [29,30]. These sequences allow the spliceosome to recognize introns and to distinguish introns from exons. The 5’ SS contains a GU dinucleotide sequence, whereas the 3’ SS contains an AG dinucleotide in majority of introns. However, these two sequences cannot be used to identify an intron. A variable stretch of pyrimidine nucleotides, which is known as the polypyrimidine tract, is situated between the 3’ SS and the BPS. The polypyrimidine tract defines the 3’ SS and recruits splicing factors to the 3’ SS and BPS. The BPS initiates a nucleophilic attack on the 5’ SS, making a branch-like structure. The BPS includes a conserved adenosine nucleotide at which to initiate the splicing process [31,32,33].

Five snRNP complexes (U1, U2, U4/U6, and U5) assemble and recognize on each intron to form a catalytically active spliceosome [4,5,6,7,8,9,30]. The spliceosome functions in a dynamic and complex cycle of assembly, reaction, and disassembly (Figure 1). The earliest steps of spliceosome assembly are the recognition of the 5’ SS and BPS by the U1 and U2 snRNPs through base-pairings. The first complex (complex E) is achieved by the binding of: (i) U1 snRNP to the 5’ SS; (ii) splicing factor 1 (SF1) to the BPS; (iii) U2 small nuclear RNA auxiliary factor 2 (U2AF2) to the polypyrimidine tract; and (iv) U2AF1 to the 3’ SS. Next, the formation of complex E enhances the recruitment of U2 snRNP to the BPS and induces the formation of complex A. The U2 snRNP consists of splicing factor 3A (SF3A), splicing factor 3B (SF3B), and a 12S RNA subunit, with SF3B1 responsible for the binding to the BPS [34]. In addition, many proteins, as well as the main spliceosome factors, are important for the determination of splicing at a particular site. Members of the serine/arginine (SR) family of proteins are involved in splicing through the detection of specific sequences in pre-mRNA, ESEs, and ISEs [31]. Generally, SR proteins interact with these sequences to enhance splicing from nearby splice sites through the recruitment of the U1 snRNP and U2AF proteins to 5’ SS and 3’ SS, respectively. Heterogeneous nuclear ribonucleoproteins (HNRNPs) suppress splicing through their association with exonic and intronic splicing silencers (ESSs and ISSs) [35]. 

After the preassembled U4/U6.U5 tri-snRNP complex is recruited to the splicing site, the U1/U4 snRNPs are released to form the catalytically activated complex B (complex B*). Further conformational rearrangements lead to the formation of the catalytic step 1 spliceosome (complex C) [4]. The catalysis of splicing consists of two major sequential transesterification reactions, catalyzed by complex B and complex C, respectively. These esterification processes excise the intron as an intron lariat and ligate the proximal and distal exons to produce mature mRNA. Thus, the composition of the cis-acting regulatory sequences and RNA-binding proteins that recognize and bind to these sites allow the adoption of various alternative splicing patterns [33].

## 4. Altered mRNA Splicing in Cancer

Unexpected, recent genomic sequencing analysis has revealed that the dysregulation of pre-mRNA splicing is involved in cancer initiation, maintenance, and progression [1,36]. Genetic mutations in cancer cells associated with mis-splicing can be classified into two categories [37]: “cis-acting mutations” that occur within the mRNA sequence that is being spliced and therefore influencing splicing; and “trans-acting mutations”, in which there are changes in the expression level or mutations of splicing factors. Cis-acting mutations have been found close to the 5’ SS, 3’ SS, BPS, and splicing enhancer or silencer elements [38]. Mutations affecting splicing are found within introns or exons and contain both synonymous and nonsynonymous mutations [39]. Such mutations are commonly known to inactivate tumor suppressor genes in the development of cancer. For example, recurrent somatic mutations in adenomatosis polyposis coli tumor suppressor *(APC)* result in exon skipping or the creation of a new splice site [40,41]. Similarly, recurrent synonymous mutations within tumor protein 53 *(TP53)* were found in close proximity to splice sites, leading to intron retention or activation of a cryptic splice site to produce a frameshifted mRNA [36]. Recurrent somatic mutations impacting on trans-acting spliceosome components were first reported in hematopoietic malignancies such as myelodysplastic syndromes (MDS) [42] and chronic lymphocytic leukemia (CLL) [43]. These mutations were found predominantly in both SF3B1 and U2AF1, which participate in core spliceosomal components that recognize the 3’ SS and serine/arginine rich splicing factor 2 (SRSF2), an SR protein [44,45,46,47,48,49]. Mutations in *SF3B1* [43], *U2AF1* [47], or *SRSF2* [49] change mRNA splicing patterns in a manner distinct from loss of function. These mutations induce cryptic 3’ SS selection through the reduction of branch point fidelity (*SF3B1* mutation) [48] or RNA-binding preferences in a sequence specific manner (*SRSF2* mutation) [49]. Mutations in *SF3B1*, *U2AF1*, and *SRSF2* were found as heterozygous point mutations at specific amino acids and occur in a mutually exclusive manner to one another [50]. Mutations in these splicing factors have also been found in solid tumors. These mutations contain *SF3B1* mutations in uveal melanoma (15–19%), pancreatic ductal adenocarcinoma (4%), and breast cancer (2–4%), as well as *U2AF1* mutations in lung adenocarcinoma (3%) [44,46,50]. In addition, because cells with a mutant splicing factor are sensitive to genetic or pharmacologic modulation of splicing, targeting splicing may be an effective treatment strategy for cancer [51].

## 5. Splicing Factor Drives Prostate Cancer Progression

The alteration of splicing activity by mutations induces severely abnormal development and tumorigenesis [3]. However, these mutations have rarely been identified in prostate cancer, although several large scale genomic sequence analyses of primary and metastatic prostate tumors have been conducted [52]. Previous studies [52,53] have identified altered signals in prostate cancer such as TP53, androgen, and Forkhead box protein A1 (FOXA1) signaling, DNA repair, and phosphoinositide3-kinase (PI3K)–AKT signaling. Alternatively, prostate cancer is classified by ETS transcription family fusions or mutations in Speckle-type POZ protein (SPOP). A recent larger study identified that splicing pathway was altered in 4% of prostate tumors [54]. As observed in other cancer types, *SF3B1* (1.1%) and *U2AF1* (0.5%), which are associated with the recognition of 3’ SS, were mutated. However, the frequency of mutation in these factors is relatively low compared with other types of cancer [37,42,43,44,45,46,47]. However, several reports have emphasized the importance of *AR* splicing in prostate cancer progression [55]. Altered splicing machinery would result in a dysregulated *AR* splicing process. *AR-V7* mRNA synthesis requires the removal of intron sequences through an RNA recognition process. The spliceosomal complex excises the 3’ SS and ligates exon 3 with exon 3B by recognizing this exon [55,56]. Notably, recent functional and clinical analyses revealed that various splicing factors are highly expressed in CRPC tissue, indicating that the splicing system plays an important role in prostate cancer progression for reasons other than mutation, for example, possibly through overexpression [22,57]. The enhanced expression of splicing factors would promote their recruitment to pre-mRNA, facilitating this dynamic and reversible mRNA splicing process. Therefore, I have summarized these representative reports showing several new driving forces for prostate cancer progression and enhanced AR signaling.

### 5.1. Splicing Factor, Proline- and Glutamine-Rich/ Non-POU Domain-Containing Octamer-Binding Protein (PSF/NONO)

**Figure 3:**
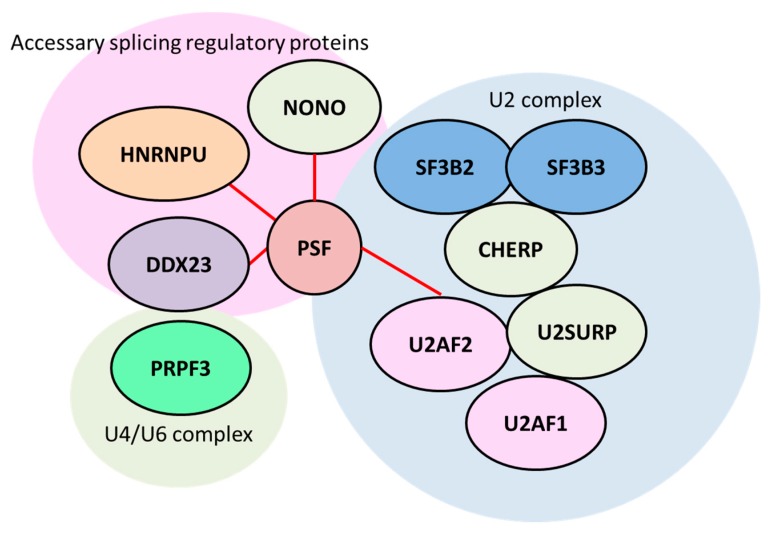
Splicing factor proline- and glutamate-rich (PSF) governs splicing factor complexes in advanced prostate cancer that are involved in AR and cancer-related gene expression. RNA-precipitation and sequencing studies (RIP-seq) revealed that PSF binds to a variety of splicing factors that are uniformly induced in metastatic castration-resistant prostate cancer (CRPC) tissues. The identified PSF-regulated splicing factors that are involved in AR and AR-V7 production are shown. The red line indicates protein–protein interaction validated by immunoprecipitation analysis [57]. HNRNPU: heterogeneous nuclear ribonucleoprotein U. PRPF3: pre-mRNA processing factor 3. CHERP: calcium homeostasis endoplasmic reticulum protein. DDX23: DEAD (Asp-Glu-Ala-Asp) box (DDX) 23. NONO: non-POU domain-containing octamer-binding protein.

A recent study showed a role of a splicing factor, proline- and glutamine-rich (PSF/SFPQ), in the progression of prostate cancer by using the deep-sequence based approach of clinicopathological analysis [57]. High PSF protein expression was observed in a subset of tumor samples and higher expression of PSF was associated with cancer-specific survival after surgery and the PSA-free survival after hormone-therapy. In addition, *PSF* mRNA expression was significantly elevated in metastatic or advanced prostate cancer samples, suggesting that PSF expression was associated with prostate cancer progression. Using an in vivo model of CRPC, knockdown of PSF reduced the growth of tumors in mice castrated after tumor development. Notably, the study presented a new mechanistic insight, with the identification of global RNAs bound with PSF. Long non-coding (lnc) RNAs, such as androgen-regulated lnc RNA, C-terminal binding protein 1 antisense *(CTBP1-AS)* [58], and a prostate cancer-associated lncRNA, switch/sucrose non-fermentable (SWI/SNF) complex antagonist associated with prostate cancer 1 *(SchLAP1)* [59], are positively regulated by PSF in prostate cancer cells. It was also found that various spliceosome genes are uniformly upregulated in metastatic CRPC tissues [57]. Furthermore, the main targets of PSF at the RNA level are these spliceosome genes (Figure 3). Widespread upregulation of the splicing pathway in CRPC could affect the splicing complexes in cancer. In addition, by regulating spliceosome gene expressions and cooperating with those factors at protein level, PSF may control the activation a broad range of oncogenic pathways. This study indicates the importance of PSF in the regulation of splicing machinery in aggressive prostate cancer [57]. These analyses provide an intriguing insight into the mechanism of splicing machinery in cancer progression [37].

**Figure 4:**
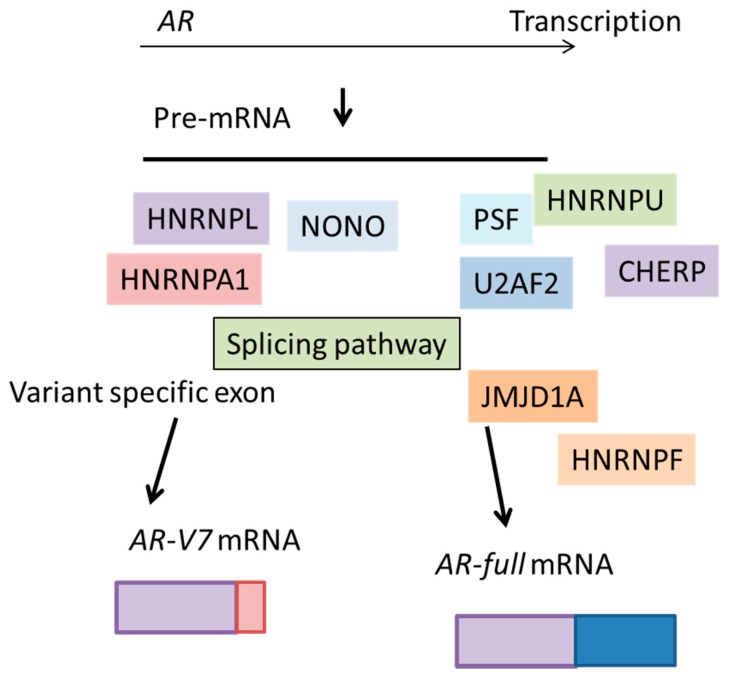
The essential role of various splicing factors for AR-V7 production. Pre-mRNA of *AR* is spliced by splicing factors upregulated in advanced prostate cancer. In prostate cancer, many splicing factors are transcriptionally induced to modifying the gene expression profile and induce cancer progression. Recently reported representative splicing factors are shown. JMJD1A: Jumonji domain containing 1A. HNRMPL: heterogeneous nuclear ribonucleoprotein L. HNRNPA1: heterogeneous nuclear ribonucleoprotein A1. HNRMPF: heterogeneous nuclear ribonucleoprotein F.

It is notable that one of the most important targets of PSF involved in CRPC is AR, because AR and AR-V7 drives the hormone-refractory state [55,56,60]. Enhanced association of *AR* transcript with PSF was observed in CRPC model cells compared with hormone-dependent cancer cells [58]. PSF regulates *AR* splicing process and promotes production of *AR* and its variants at the mRNA level. PSF forms a complex with other RNA-binding proteins and non-POU domain-containing octamer-binding protein (NONO) [61] to promote splicing events of *AR* transcripts and AR-V7 production (Figure 4). Through immunoprecipitation and western blotting analysis, PSF was shown to interact with these spliceosome components, and be responsible for the complex formation of spliceosome. A RIP assay also demonstrated that these factors bound to the intronic region of *AR* transcripts and acted as an integrator for the PSF-mediated splicing of *AR* transcripts. Thus, PSF orchestrated its target splicing factors at the protein levels to form a complex for splicing and protein expression in CRPC. These functions of RNA-binding proteins could be effective for enhancement of the expressions of prostate cancer-associated genes, such as full-length AR and AR-V7. The expression of these important components, such as CHERP, U2AF2 and HNRNPU [62,63,64,65,66], are enhanced by PSF binding. Another report showed that the recruitment of U2AF2 (also called U2AF65) to the 3’ splice site of *AR-V7* with SRSF1 was enhanced by ADT to produce increased expression of AR-V7 [60]. Collectively, these results suggested that PSF functions as the “commander” of splicing machinery for prostate cancer progression and the AR.

### 5.2. Heterogeneous Nuclear Ribonucleoprotein (HNRNP) Family Members

A genome-wide clustered regularly interspaced short palindromic repeat (CRISPR) screen has identified the essential genes for prostate cancer growth [67]. This study demonstrated that heterogeneous nuclear ribonucleoprotein L (HNRNPL) was the most important splicing factor. Comprehensive analysis of HNRNPL-dependent alternative splicing processes in prostate cancer cells identified direct HNRNPL-regulated signals. HNRNPL binds preferentially to cytosine adenine (CA)-repeats or CA-enriched RNA motifs in introns, and the 3’ untranslated region (UTR) regions of genes. Similar to other HNRNP family proteins function in alternative splicing, HNRNPL enhances and represses alternative splicing of different targets [35]. Overall, the identification of AR as an HNRNPL-regulated splicing target gene at the RNA level is of interest (Figure 4). As described above, HNRNP family members have important roles as enhancers or repressors of splicing processes [68,69]. It has been shown that HNRNP A1 upregulates Lin28 [70], NKKB2/p52 [68], and c-Myc [68], and activates AR-V7. The knockdown of HNRNPA1 downregulated AR-V7 and resensitized enzalutamide-resistant cells to enzalutamide [68]. In addition, *HNRNPH1* transcripts negatively regulated by *miR-212* were associated with the expression of AR and AR-V7 in prostate cancer cells. HNRNPH1 physically interacts with AR and steroid receptor coactivator-3 (SRC-3), and activates androgen-regulated genes in both a ligand-dependent and -independent manner. Silencing of HNRNPH1 sensitized prostate cancer cells to bicalutamide and inhibited prostate tumor growth in vivo [71]. These findings suggested that HNRNP family proteins cooperate in the splicing event of AR in CRPC development. Moreover, the HNRNPL-regulated splicing targets were prominently associated with the overexpressed gene signatures in prostate cancer, indicating that these HNRNPL targets may collectively contribute to cancer progression [67]. 

HNRNPL is also involved in circular RNA (circRNA) formation [72,73]. Trans-factor binding or direct complementary sequence pairing at flanking introns may increase circRNA formation by bringing back spliced exon ends into close proximity [67]. Notably, the HNRNPL-regulated circRNA genes are relevant to prostate cancer progression, which is suggestive of the potential role of circRNAs produced from these genes [67]. 

### 5.3. Jumonji Domain Containing 1A (JMJD1A)

The histone demethylase, Jumonji domain containing 1A (JMJD1A), was originally found to function as a key coactivator for AR through epigenetic regulation of H3K9 methylation marks [74,75]. Moreover, JMJD1A can also act to demethylate non-histone proteins in addition to histone H3K9 [76,77]. Another JMJ protein, JMJD6, an arginine demethylase and lysine hydroxylase, can regulate the alternative splicing through the hydroxylation of U2AF2 [78], suggesting the possibility of JMJ proteins as splicing factors. Indeed, JMJD1A was reported to be involved in the splicing of *AR-V7* in prostate cancer cells [79] (Figure 4); mechanistically, HNRNPF was shown to interact with JMJD1A to promote this splicing event [79]. HNRNPA1 and HNRNPF function together to enhance the splicing events of some pre-mRNAs [68,79,80,81]. The JMJD1A–HNRNPF complex may interact with HNRNPA1-mediated *AR-V7* splicing. The expression of JMJD1A was significantly associated with that of AR-V7 in prostate cancer tissue. Thus, the level of JMJD1A or factors that regulate the JMJD1A–HNRNPF interaction would promote the alternative splicing of *AR-V7* in prostate cancer. Because increased levels of AR and AR-V7 are important for CRPC progression [21,56], current therapeutic strategies aimed at targeting AR are unlikely to have significant effects in prostate cancers expressing ARV7. Therefore, the finding that JMJD1A regulates the activity of AR and the generation of AR-V7 suggests that future development of specific JMJD1A inhibitors should be considered [79].

### 5.4. Other Splicing Factors Associated with AR-V7 Production

Another RNA-binding protein, Src-associated in mitosis 68 KDa protein (Sam68), is involved in an alternative splicing process by 3’-end formation of mRNA [82]. Sam68 is upregulated in prostate cancer tissues, promotes *AR-V7* splicing through direct binding to its transcripts and U2AF2 and SRSF1, which modulates the 3’ SS of AR-V7. It also interacts with AR-V7 protein and coactivates AR-V7 target genes, including UBE2C. Moreover, DEAD (Asp-Glu-Ala-Asp) box (DDX) RNA helicases such as DDX39A, B [83], lncRNA metastasis associated in lung adenocarcinoma transcript-1 *(MALAT1)* [84], and several kinases, such as Aurora A [85] and AKT [86], are also involved in AR-V7 production through their interacting with splicing factors. *MALAT1* is regulated by suppressive androgen responsive element (ARE) in the promoter. Therefore, treatment with enzalutamide, an AR antagonist, enhances the expression. Increased *MALAT1* associates with SRSF1 (also known as SF2) to promote AR-V7 production. *MALAT1* knockdown and the enhanced AR-V7 degradation suppressed enzalutamide-resistant prostate tumor growth in vivo [84].

## 6. Splicing Factors Involved in the Development of Neuroendocrine Prostate Cancer

A subtype of castrate-resistant prostate cancer, called treatment-induced neuroendocrine prostate cancer (NEPC), has occurred owing to the widespread use of AR blockers [87]. NEPC has been reported to be found in approximately 25% of patients with prostate cancer who received AR deprivation therapies. Patients with NEPC and small cell carcinoma can survive for only a short period after diagnosis. Although systemic chemotherapy regimens are used, no targeted therapy has been approved for patients with NEPC [88]. Whole exome sequencing studies have demonstrated that clinical NEPC and adenocarcinoma prostate cancer (AdPC) tumors have a similar gene mutation landscape. In addition, in vitro experiments demonstrated an AdPC to NEPC cell lineage switch by a transdifferentiation mechanism [89]. This NEPC transdifferention event was mediated by actions of transcriptional repressor of neuronal genes RE-1 silencing transcription factor (REST) [90] or epigenetic modulators, including enhancer of zeste, polycomb repression complex 2 (EZH2) [91]. These results emphasize that many factors have an important role in NEPC progression.

It is of note that a splicing event is an essential part of this mechanism. Serine/arginine repetitive matrix 4 (SRRM4) strongly drives NEPC development [92]. SRRM4 induces several neuroendocrine markers, such as synaptophysin (SYP), CD56, and chromogranin A (CHGA), which are commonly used for NEPC diagnosis [89,92]. Reports propose that SRRM4 may be a more reliable early diagnostic marker for NEPC than those previously used. Immunohistochemistry studies demonstrated that SRRM4 has a negative predictive power for excluding NEPC tumors, and that SRRM4-positivity was more sensitive than CD56, SYP, or CHGA [92]. SRRM4 promotes alternative splicing and the expression of neural-specific transcripts. Thus, the expression of SRRM4 is associated with the neuroendocrine phenotype in CRPC tumors. It was shown that the splicing of REST occurs in AR neuroendocrine patient-derived xenograft (PDX) models, and the splicing in of 62 bp into *REST* mRNA inactivates REST, implying that SRRM4 induced NEPC independently from the REST-mediated mechanism [92]. SRRM4 induced dramatic neuronal morphological changes that accelerate cell proliferation in AR-negative prostate cancer cells [93]. In addition, SRRM4 activates pluripotency gene networks associated with stem-cell like differentiation [93]. One target of particular interest is SRY-Box 2 (SOX2), which promotes lineage plasticity and anti-AR therapy resistance in TP53- and retinoblastoma 1 (Rb1)-deficient prostate cancer [94,95]. These results showed the importance of SRRM4 for lineage switching in NEPC development, through the activation of a neuronal-specific splicing and pluripotency gene network [96].

## 7. Conclusions

There is an abundance of evidence that the alternative splicing pathway is essential for gene regulation in cells. The dysregulation of splicing is involved in many types of cancer, including prostate cancer. In this review, a number of cancer-specific spliceosomal factors have been discussed, including PSF and SRRM4. From the analysis of clinical data, it has been demonstrated that a variety of splicing factors are upregulated in metastatic prostate cancer tissues, indicating the possibility that enhanced splicing ability induces the diversity of gene functions and drives the progression of prostate cancer into CRPC. There is a pressing need to expand research into alternative splicing in the context of many other important diseases. Moreover, understanding the regulation of alternative splicing should provide a new pathway for the development of novel therapeutic strategies.
